# Bone mineral density is associated with vitamin D related rs6013897 and estrogen receptor polymorphism rs4870044: The Tromsø study

**DOI:** 10.1371/journal.pone.0173045

**Published:** 2017-03-02

**Authors:** Ieva Martinaityte, Rolf Jorde, Nina Emaus, Anne Elise Eggen, Ragnar Martin Joakimsen, Elena Kamycheva

**Affiliations:** 1 Tromsø Endocrine Research Group, Department of Clinical Medicine, UiT The Arctic University of Norway, Tromsø, Norway; 2 Division of Internal Medicine, University Hospital of North Norway, Tromsø, Norway; 3 Department of Health and Care Sciences, UiT The Arctic University of Norway, Tromsø, Norway; 4 Epidemiology of chronic diseases research group, Department of Community Medicine, UiT The Arctic University of Norway, Tromsø, Norway; Leeds Beckett University, UNITED KINGDOM

## Abstract

**Background:**

Bone mineral density (BMD) is determined by bone remodeling processes regulated by endocrine, autocrine and genetic mechanisms. Thus, some studies have reported that BMD is associated with single nucleotide polymorphisms (SNPs) associated with vitamin D receptor (*VDR*), serum 25(OH)D levels and estrogen receptor 1 (*ESR1*), but without consensus. Therefore, we aimed to map and compare the risk genotypes for forearm and total hip low BMD.

**Methods and findings:**

Data were derived from a population-based study in northern Norway; the Tromsø Study. Distal forearm BMD was measured with a single x-ray absorptiometric device, while total hip BMD was measured with a dual-energy x-ray absorptiometric device. There were 7,317 and 4,082 successful analyses of distal forearm and total hip BMD, respectively, and at least one SNP of interest. We evaluated plausible BMD modulating factors and associations of BMD and SNPs related to vitamin D metabolism (*FokI*, *Cdx2*, *BsmI*, rs2298850, rs10741657, rs3794060, rs6013897), *ApaI-BsmI-TaqI* haplotypes and *ESR1* SNP rs4870044.

**Results:**

Age, BMI, physical activity and smoking were significantly associated with BMD. In a linear regression model with adjustment for age and gender and with the major homozygote as reference, rs6013897 had a standardized beta coefficient (*β*) of –0.031 (*P* = 0.024) for total hip BMD. *β* for *ESR1* SNP rs4870044 was –0.016 (*P* = 0.036) for forearm BMD and –0.034 (*P* = 0.015) for total hip BMD. The other SNPs nor serum 25(OH)D were significantly associated with BMD.

**Conclusions:**

Both forearm and total hip BMD were associated with ESR1 SNP rs4870044. Of the vitamin D–related genes, only *CYP24A1* gene rs6013897 was associated with total hip BMD, but the association was weak and needs confirmation in other studies. Serum 25(OH)D was not associated with BMD in our population, probably due to the generally sufficient vitamin D levels in the population.

## Introduction

Osteoporosis, characterized by low bone mineral density (BMD), is a global health problem [1]. BMD is a multifactorial trait and in 50–90% of cases is possibly determined by genetic factors [2,3]. Every new identified factor may improve the prevention and treatment of bone loss, with future individual-tailored approaches.

Vitamin D action in bone homeostasis is explained by the modulation of gene expression and the activation of second-messenger systems when 1,25-dihydroxyvitamin D (1,25(OH)D) binds to and activates the vitamin D receptor (VDR) [4,5]. VDR is found in all tissues involved in vitamin D–related calcium homeostasis [4] and bone maintenance [6]: the parathyroid gland, small intestine, kidney and bone [4]. Consequently, vitamin D levels [7] and defects or changes in VDR function [8] are associated with bone mineralization alterations.

Recently, BMD associations with VDR-related single nucleotide polymorphisms (SNPs) have been reported [2]. The risk of osteoporosis and the response to vitamin D may vary due to these genetic variations, although the genetic effects may be small. Nevertheless, the *VDR* SNP *Cdx2* genotype (rs11568820, g.47908762C>G (chr12, GRCh38.p7) seems to be associated with risk of fracture [9] as are some *VDR* SNP haplotypes (*BsmI-ApaI-TaqI*) [10]. However, Uitterlinden *et al*.’s meta-analysis reported no clear association between *VDR* SNPs and BMD [9].

Though the genetically determined heritability of serum 25(OH)D is reported to be ~43% [3], *VDR* SNPs do not seem to determine serum 25(OH)D levels [11,12]. The associations between other vitamin D related SNPs and serum 25(OH)D levels are inconsistent and with potentially limited influence under the physiological conditions [12,13].

Another active substance, estrogen, is known to be bone-protective by acting via the estrogen receptor 1 (ESR1) expressed in osteoblasts and osteoclasts [14,15]. Several SNPs in the 6q25 locus related to ESR1 are reported to be associated with total hip BMD [16], but no studies regarding forearm BMD and *ESR1*-associated SNP rs4870044 (g.151580274C>T (chr6, GRCh38.p7)) have been published.

As regional and interracial genetic differences may be present and mapped risk genes may be useful as therapeutic targets, we aimed to investigate whether SNPs associated with VDR, ESR1 and vitamin D levels could be risk factors for low forearm and total hip BMD in 7,317 and 4,082 Norwegians respectively.

## Materials and methods

### Study population

The Tromsø Study initiated in 1974 is a longitudinal, population-based multipurpose Norwegian study conducted every 6–7 years in the Tromsø municipality. For the fourth survey (Tromsø 4) in 1994–1995, all individuals age 25 years or older were invited; 77% (or 27,158 subjects) participated [17]. Distal forearm BMD was successfully measured in 7,828, as 120 subjects were excluded due to movement artifacts or unfulfilled informed consent [18].

Total hip BMD was successfully measured in 4,605 subjects in the fifth survey (Tromsø 5) in 2001–2002 (695 subjects were excluded due to ineligible scans) [19]. The participants of Tromsø 5 were either the individuals with BMD measurements from Tromsø 4 (7,386 subjects), or the randomly selected group age 30–75 years (2,967 subjects) with an attendance rate of 79% (or 8,130 subjects).

In Tromsø 4, 11,752 subjects (selected based on subsequent endpoints of interest or as controls) were successfully genotyped as previously described in detail [13].

Thus, 7,317 subjects (4,197 women and 3,120 men) with both successful genetic analyses and distal forearm BMD measurements from Tromsø 4 as well as 4,082 subjects (2,440 women and 1,642 men) with both the successful SNP analyses and total hip BMD measurements from Tromsø 5 were included in the analyses in the present study.

### Questionnaires

Information on possible BMD modulating factors [1,7] was collected from self-administered questionnaires in Tromsø 4 and 5. Self-reported use of drugs and supplements was assessed through questionnaires and also a written list of brand names of drugs used on regular basis checked by health personnel at the study site (in Tromsø 4 only in subjects aged 55–74 years, and 5.5% of participants >74 years). The data included current or previous use of estrogen (both systemic and local, as the use of local estrogen does not eliminate the systemic effect [20]), systemic corticosteroids, thiazide diuretics, insulin, other antidiabetic drugs, bisphosphonates, vitamin D (in the form of cod liver oil or vitamin D supplement use) and calcium supplementation. In addition, self-reported early menopause (defined as age <47 years and ≥12 months of continuous amenorrhea), previous or current cancer, diabetes, osteoarthritis, malabsorption (self-reported ulcer-related surgery), physical activity and current or previous daily smoking were also registered. Physical activity was defined as the presence or absence of light or hard physical activity for an average of 1 h per week during leisure time.

### Body measurements

BMD measurements of the distal forearm were performed once only in Tromsø 4 with a single x-ray absorptiometric device (DTX-100; Osteometer MediTech, Inc., Hawthorne, CA, USA) in the radius and the ulna from the 8-mm point (the point where the ulna and the radius are separated by 8 mm) and 24 mm proximally. The non-dominant arm was measured except when it was ineligible due to foreign bodies or wounds [21].

Total hip BMD was measured once only in Tromsø 5 with a dual-energy x-ray absorptiometric device (GE Lunar Prodigy, LUNAR Corporation, Madison, WI, USA) [22]. The means of the right and left hip scans were used for the analyses when available; otherwise, one-side hip scans were used.

Due to the quality control and assessment routines described previously [23], the data on forearm BMD were adjusted to improve precision by correcting the artifacts and long-term drift throughout each survey and between surveys (using the European forearm phantom). All scans were performed and reviewed by specially trained technicians in both Tromsø 4 and 5 who followed standardized protocols [21,22].

In both surveys, the subjects’ weight and height were measured while they wore light clothing without shoes. Body mass index (BMI) was calculated as weight divided by height squared (kg/m^2^).

### Laboratory methods

Non-fasting blood samples were obtained for measurement of factors related to bone mass (hyperparathyroidism, vitamin D deficiency [7,19], hyperthyroidism [24,25], chronic kidney disease [26]). Serum parathyroid hormone (PTH; Tromsø 4 only; reference range 1.1–6.8 pmol/l for subjects ≤50 years and 1.1–7.5 pmol/l for subjects >50 years) was measured on an Immulite analyzer (Diagnostic Products, Los Angeles, CA, USA) based on a two-site chemiluminescent immunometric assay with a coefficient of variation (CV) of 6–8% in the actual range. Serum 25-hydroxyvitamin D (25(OH)D) (Tromsø 4 only; reference range 50–90μmol/l for women and 60–100μmol/l for men) was measured. Sera were stored at –70°C, thawed in 2008 and analyzed with an electrochemiluminescence immunoassay in an automated clinical chemistry analyzer (Modular E170, Roche). Serum 25(OH)D values were recalibrated according to the Vitamin D Standardization Program (VDSP) protocol based on the liquid chromatography-tandem mass spectrometry (LC-MS/MS) method [27]. Serum thyroid-stimulating hormone (TSH; Tromsø 4 and 5; reference range 0.20–4.00 mIU/l) was analyzed with an automated clinical chemistry analyzer (Immulite 2000; DPC, Los Angeles, CA, USA). As a covariate, TSH was divided into three groups: <0.49 mIU/l, 0.49–4.56 mIU/l and >4.56 mIU/l based on previous findings in the Tromsø population [25]. Serum creatinine (Tromsø 4 and 5; reference range 55–100 μmol/L for women and 70–100 μmol/L for men) and serum calcium (Tromsø 4 and 5; reference range 2.20–2.60 mmol/L) were analyzed using a Hitachi Model 917 analyzer with reagents from Boehringer Mannheim (Mannheim, Germany).

DNA was prepared with the manual isolation method from whole blood samples collected during the subjects’ first visit in Tromsø 4. Genotyping was performed with the KBioScience Allele-Specific Polymorphism (KASP) SNP genotyping system as previously described [13].

### Selection of SNPs associated with BMD and vitamin D

As defects or changes in VDR function [8] are associated with BMD, we selected the SNPs associated with VDR function and earlier reported to be associated with BMD or fracture risk [2,9]. Therefore we considered the following eight VDR-associated SNPs: *FokI* (rs2228570/rs10735810, g.47879112A>T (chr12, GRCh38.p7)), *BsmI* (rs1544410, g.47846052C>G (chr12, GRCh38.p7)), *TaqI* (rs731236, g.47844974A>T (chr12, GRCh38.p7), *ApaI* (rs7975232, g.47845054C>G (chr12, GRCh38.p7)), *Cdx2* (rs11568820)), rs7968585 (g.47838310C>T (chr12, GRCh38.p7)), rs3782905 (g.47872384G>C (chr12, GRCh38.p7)) and rs2239179 (g.47863983T>A (chr12, GRCh38.p7)).

Levels of 25(OH)D [7] are also associated with BMD, therefore we included the SNPs which earlier had been reported with the highest difference in mean serum 25(OH)D between major and minor homozygotes in the vitamin D binding protein (*DBP*) gene (rs2298850, g.71748550G>C (chr4, GRCh38.p7)), the gene that encodes for 25-hydroxylase (*CYP2R1*) involved in the conversion of vitamin D into 25(OH)D (rs10741657, g.14893332A>T (chr11, GRCh38.p7)), the 7-dehydrocholesterol reductase/NAD synthetase 1 gene (*NADSYN*) responsible for the availability of 7-dehydrocholesterol in the skin (rs3794060, g.71476633C>T (chr11, GRCh38.p7)) and the gene that encodes for 24-hydroxylase (*CYP24A1*) involved in the degradation of 25(OH)D and synthesis of 1,25(OH)D (rs6013897, g.54125940T>A (chr20, GRCh38.p7)) [13].

Finally, we included the rs4870044 SNP in locus 6q25 close to the *ESR1* gene as this SNP has been strongly associated with hip and lumbar spine BMD [16,28] but has not been studied regarding forearm BMD.

### Statistical analyses

Statistical analyses were undertaken with IBM SPSS Statistics 22 (SPSS Inc., Chicago, IL, USA) to test the following hypotheses:

- Hypothesis 1. Forearm and total hip BMD in the Tromsø population is associated with modulating factors of BMD.
- Hypothesis 2. Serum 25(OH)D-related SNPs are associated with forearm and total hip BMD.
- Hypothesis 3. The *ESR1* SNP rs4870044 is associated with forearm and total hip BMD.

Distribution of the continuous variables, including BMD and serum 25(OH)D, was evaluated with skewness, kurtosis and visual inspection of histogram and Q-Q plots and found to be normal. As a covariate, serum PTH was divided into quartiles [19].

To test hypothesis 1, we used forearm or total hip BMD as a dependent variable, and the plausible modulating factors of BMDs as covariates in the linear regression model. Height was included to adjust for the forearm BMD measurement. Missing values for self-reported parameters such as use of drugs and diseases/conditions were interpreted as negative values. Early menopause and use of estrogen were included as covariates; therefore, men and women were analyzed separately.

To test hypotheses 2 and 3, the genotype frequencies were evaluated with a chi-square test for Hardy-Weinberg equilibrium [29]. Linkage disequilibrium (LD) was calculated using SNP Annotation and Proxy Search [30] based on International HapMap Project data.

Trends for categorical variables across the selected SNPs were evaluated with the chi-square test with linear-by-linear association and for the continuous variables with linear regression adjusted for age and gender. When serum 25(OH)D was included, additional adjustments for season (months, using dummy variables) were performed.

For significant linear trends for BMD across the SNP, the regression model was adjusted for other variables significantly associated with BMD. Contrasts between genotypes were calculated with univariate linear model, adjusted for age and gender.

For the *VDR* SNP haplotypes *BsmI*-*ApaI*-*TaqI*, we tested the interactions between the *BsmI*, *ApaI* and *TaqI* genotypes with a linear regression.

The data are shown as mean ± standard deviation (SD). All tests are presented two-sided, with the standardized beta coefficient in the regression analyses. A *P* value of less than 0.05 was considered statistically significant. The data are presented without Bonferroni correction for multiple testing unless specified otherwise.

### Ethics

The study was approved by the Regional Committee for Medical and Health Research Ethics (REK Nord) (reference 2010/2913-4). Only participants with valid written consent were included.

## Results

The baseline characteristics of the entire Tromsø 4 and Tromsø 5 populations and the groups of subjects with valid BMD measurements of the forearm and hip and at least one successful genotyping are shown in Table 1 and S1 Table.

**Table 1:** The baseline characteristics of the entire study population and subjects with valid BMD measurements of the forearm in Tromsø 4 and hip in Tromsø 5, and at least one successful SNP analysis*. The Tromsø Study.

	Entire Tromsø 4 population**	Genotyped and BMD measured subjects in Tromsø 4	Entire Tromsø 5 population**	Genotyped and BMD measured subjects in Tromsø 5
N	26,956	7,317	8,039	4,082
Age (years)^a^	46.9 ± 15.1	58.9 ± 10.4	59.7 ± 14.1	64.6 ± 9.6
Sex (% female)	52.5	57.4	56.8	59.8
Distal forearm BMD ^b^	0.47 ± 0.09	0.46 ± 0.09	NA	NA
Total hip BMD	NA	NA	0.95 ± 0.15	0.95 ± 0.15
BMI (kg/m^2^)	25.2 ± 3.9	25.9 ± 4.0	26.7 ± 4.3	26.8 ± 4.1
Height (cm)	168.7 ± 9.3	167.6 ± 9.2	168.0 ± 9.4	167.1 ± 9.1
Serum PTH (pmol/l) ^b^	2.79 ± 1.43	2.8 ± 1.4	NA	NA
Standardized serum 25(OH)D (nmol/L) ^b^	54.5 ± 11.6	54.5 ± 11.6	NA	NA
Serum creatinine (μmol/L) ^b^	67.3 ± 16.2	67.4 ± 16.5	70.6 ± 17.2	70.7 ± 16.8
Serum calcium (mmol/L)	2.38 ± 0.10	2.39 ± 0.10	2.36 ± 0.09	2.36 ± 0.09

^a^Age by the end of 1994 for Tromsø 4, and age by the end of 2001 for Tromsø 5.

^b^In Tromsø 4, information attained only in those who attended the second visit in 1994–1995, N from 2,903 to 7,872.

* SNPs of interest include the SNPs presented in Table 2 (*FokI*, *Cdx2*, *BsmI*, rs2298850, rs10741657, rs3794060, rs6013897 and rs4870044).

**The number of subjects differs from the original number in description as the data in the table are presented based on the data available to the author.

NA: not available.

### Variables associated with forearm BMD

In 1,674 women, BMI, height, serum creatinine, serum calcium, use of vitamin D and estrogen had a significant positive association with forearm BMD. Age, serum PTH, self-reported cancer, early menopause and use of calcium were negatively associated with forearm BMD in the same group (S2 Table).

In 1,276 men, BMI and height were positively associated with forearm BMD, while age, smoking, self-reported ulcus surgery, use of insulin and systemic cortisone had a negative association (S2 Table).

Serum 25(OH)D was not significantly associated with forearm BMD in either men or women. Bisphosphonates were not included as a covariate, as there were only two users in Tromsø 4.

### Variables associated with total hip BMD

In 875 women, BMI and physical activity were positively associated with total hip BMD, while age, serum PTH, smoking and use of bisphosphonates were negatively associated with total hip BMD (S3 Table).

In 626 men, BMI and physical activity had a positive association with total hip BMD, while age, smoking and self-reported ulcus surgery were negatively associated with total hip BMD (S3 Table).

Serum 25(OH)D was not significantly associated with total hip BMD in either men or women.

### Associations between *VDR* SNPs and BMD

The *VDR* SNP *Bsml* was chosen for analyses as it is the most consistently associated with BMD [2]. The *VDR* SNPs with high or moderate LD (r^2^ ≥ 0.4) with *BsmI* were excluded (*TaqI*, r^2^ = 1.0; rs2239179 and rs7968585, r^2^ = 0.65; *ApaI*, r^2^ = 0.57; and rs3782905, r^2^ = 0.47). *FokI* and *Cdx2* were in low LD (r^2^ < 0.4) with other *VDR* SNPs and were included in the analyses.

However, in the linear regression model adjusted for age and gender, none of the three selected *VDR* SNPs (*FokI*, *Cdx2*, *BsmI*) were significantly associated with BMD (Table 2).

**Table 2:** BMD trends across the SNPs analyzed with linear regression, adjusted for age and gender in Tromsø 4 and Tromsø 5.

	Forearm BMD, Tromsø 4		Total hip BMD, Tromsø 5	
SNP	N	Standardized beta coefficient	N	Standardized beta coefficient
Rs2228570/rs10735810 (*FokI*, *VDR* SNP)	7260	–0.014	4051	–0.021
Rs11568820 (*Cdx2*, *VDR* SNP)	7257	–0.006	4049	0.001
Rs1544410 (*BsmI*, *VDR* SNP)	7213	–0.009	4027	–0.014
Rs2298850 (*DBP* SNP)	7255	0.003	4047	–0.018
Rs10741657 (*CYP2R1* SNP)	7257	–0.010	4050	–0.003
Rs3794060 (*NAD-SYN* SNP)	4897	–0.015	2510	0.002
Rs6013897 (*CYP24A1* SNP)	7233	–0.006	4039	–0.031*
Rs4870044 (*ESR1* SNP)	7281	–0.016*	4064	–0.034*

* *P* = 0.024 for rs6013897 and total hip BMD; *P* = 0.036 and *P* = 0.015 for rs4870044 and forearm or total hip BMD respectively in the linear regression model, adjusted for age and gender, not corrected for multiple testing.

Unmarked coefficients had *P* > 0.05. None of the SNPs had *P* < 0.05 after Bonferroni correction (7 × 2 × *P*).

As the haplotypes (*BsmI*-*ApaI*-*TaqI*) were associated with BMD in the Rotterdam Study [10], interactions between these SNPs regarding BMD were analyzed. However, in the present study, the interactions were not found to be statistically significant.

### Associations between serum 25(OH)D SNPs and total hip BMD

As expected [13], the four selected serum 25(OH)D SNPs were significantly associated with the serum 25(OH)D levels with the difference in the means between major and minor homozygotes ranging from (–)1.0 to 5.0 nmol/L (S4 Table).

In the linear regression model with adjustments for age and gender, a significant association with BMD was found only for the *CYP24A1* SNP rs6013897 for total hip (stand. beta coeff. –0.031, *P* = 0.024) (Table 2). The difference between the major and minor homozygote was 0.02 g/cm^2^ with *P* = 0.026 (Table 3).

**Table 3:** Comparison of means of total hip BMD across the genotypes of *CYP24A1* SNP rs6013897 in Tromsø 5, adjusted for age and gender.

*CYP24A1* rs6013897 genotypes	N	Mean±SD (g/cm^2^)	Difference (SE) (g/cm^2^)	Absolute *P-*value
Major homozygote T:T	2373	0.953±0.150	-0.006 (0.004)	0.208 (T:T vs. T:A)
Heterozygote T:A	1441	0.946±0.151	-0.015 (0.010)	0.114 (T:A vs. A:A)
Minor homozygote A:A	225	0.933±0.150	-0.021 (0.09)	0.026 (T:T vs. A:A)

Inclusion of serum 25(OH)D (N = 3,035) as a continuous or dichotomous variable (above/below the 20^th^ percentile, 43.0–50.0 nmol/L, differing according to season) or serum PTH (continuous or divided in quartiles, N = 1,773) increased *P-*value above 0.05 for the negative trend for rs6013897 and BMD. We observed the same effect after adjusting for the significantly associated variables for total hip BMD (age, sex, BMI, physical activity, serum PTH, smoking, use of bisphosphonates and ulcus surgery, S3 Table), with 1,656 subjects in the regression analysis. The estimated effect of rs6013897 adjusted only for age and gender in the selected groups mentioned above was similar.

### Associations between estrogen receptor SNP rs4870044 and BMD

Interactions between *ESR1* SNP rs4870044 and sex regarding BMD were not significant. Therefore, both sexes were analyzed together, adjusted for gender and age. We found significant linear trends across the genotypes for forearm and total hip BMD measurements (Table 2). The differences between major and minor homozygotes were 0.004 g/cm^2^ for forearm BMD and 0.01 g/cm^2^ for total hip BMD (Table 4).

**Table 4:** Comparison of means of forearm and total hip BMD across the genotypes of *ESR1* SNP rs4870044 in Tromsø 4 and 5, adjusted for age and gender.

*ESR1* SNP rs4870044 genotypes	N	Mean±SD (g/cm^2^)	Difference (SE) (g/cm^2^)	Absolute *P-*value
***Forearm BMD*, *Tromsø 4***				
Major homozygote C:C	3709	0.465±0.094	-0.003 (0.001)	0.092 (C:C vs. T:C)
Heterozygote T:C	2981	0.465±0.094	-0.003 (0.003)	0.225 (T:C vs. T:T)
Minor homozygote T:T	591	0.457±0.089	-0.004 (0.003)	0.100 (C:C vs. T:T)
***Total hip BMD*, *Tromsø 5***				
Major homozygote C:C	2038	0.956±0.150	-0.012 (0.004)	0.006 (C:C vs. T:C)
Heterozygote T:C	1699	0.944±0.151	-0.003 (0.008)	0.748 (T:C vs. T:T)
Minor homozygote T:T	327	0.940±0.144	-0.010 (0.008)	0.229 (C:C vs. T:T)

These associations between rs4870044 and total hip BMD remained statistically significant after variables associated with total hip BMD were included (N = 1,661; age, BMI, physical activity, smoking, serum PTH, self-reported ulcus surgery, use of bisphosphonates were included from S3 Table). Some variables associated with forearm BMD were sex-specific, and although interaction between the SNP and sex was not significant, we adjusted for these variables separately for each sex. In women (N = 1,792), inclusion of age, height, BMI, serum PTH, serum creatinine, serum calcium, self-reported cancer, early menopause and use of vitamin D, calcium and estrogen from S2 Table resulted in the same negative trend across the rs4870044 genotypes, with *P* = 0.084. In men (N = 2,326), rs4870044 remained negatively associated (*P* = 0.021) after adjusting for age, height, BMI, smoking, self-reported ulcus surgery and use of insulin and systemic cortisone (from S2 Table).

## Discussion

In this population-based Norwegian study, vitamin D–related SNP rs6013897 (near the *CYP24A1*; the gene that encodes 24-hydroxylase) was associated with total hip BMD, with decreasing BMD for each minor allele (A). To our knowledge, this finding is novel.

As expected, the SNP rs6013897 was associated with the serum 25(OH)D levels [12,13,31]. It was also associated with the serum PTH levels. One possible mechanism for the effect of rs6013897 on total hip BMD could be through the changes in serum 25(OH)D and serum PTH levels [32]; therefore, serum 25(OH)D and serum PTH were included as covariates in the linear regression. The associations between rs6013897 and total hip BMD had the same directions, but were no longer statistically significant. The lack of statistical significance was probably due to the decreased number of subjects (3,035 subjects were adjusted for serum 25(OH)D, and 1,773 subjects were adjusted for serum PTH). Comparing the effect of rs6013897 in 1,544 subjects (those with serum 25(OH)D measurements and full data for significantly associated variables for total hip BMD), the point estimates for the effect of rs6013897 on total hip BMD changed less than 11% when adding the selected variables into the model adjusted for age and gender. Thus, as expected due to Mendelian randomization, the effect of the SNP on total hip BMD was not due to or confounded by other variables though rs6013897 has been associated with response to vitamin D3 [33,34].

No associations were found between other vitamin D–related SNPs and forearm or total hip BMD. Activated VDR is heavily involved in bone tissue homeostasis [4,5], and associations between BMD and *BsmI* [2], as well as *BsmI-ApaI-TaqI* haplotypes, *Cdx2* and fracture risk [9,10], have been reported. However, a comprehensive meta-analysis that included 26,242 subjects did not find a clear association between *VDR* SNPs and BMD [9], in agreement with the present findings. This does not argue against an important function of the VDR in bone metabolism but more likely reflects that the SNP action on the VDR function has still not been established.

Similarly, the lack of a statistically significant association between serum 25(OH)D and BMD in the present study should be interpreted with caution, as the studied population was vitamin D sufficient; few subjects had very low serum 25(OH)D levels. In addition, only one serum 25(OH)D measurement was performed, and although there is a large degree of tracking for the serum 25(OH)D level [35], one measurement can hardly reflect lifelong exposure. In particular, it should be recalled that the peak BMD in the Tromsø population was measured in 38- to 43-year-old subjects [36], and the subjects had a mean age of 59–65 years. However, it is likely that the effect of rs6013897 on serum 25(OH)D is lifelong. Although the effect on the serum 25(OH)D level by this SNP was modest, it is a strong argument for the effect of vitamin D on bone metabolism.

This is the first report of an association between *ESR1* SNP rs4870044 and forearm BMD, to our knowledge. The rs4870044 was included in the analyses for two reasons. First, it has been firmly established that this SNP is associated with total hip BMD [16,28], and finding the same in the present study could be considered quality control. Second, this SNP has not previously been associated with forearm BMD. The finding of such a significant association was no surprise and reflects the effect of estrogen on all types of bone [14,15]. In addition, the association between rs4870044 genotypes and BMD remained significant after other BMD-associated variables were included, indicating an independent effect of this *ESR1*-associated SNP.

Low forearm BMD and subsequently increased fracture risk [37] does not lead to the same decreased health-related quality of life and increased mortality risk as hip fracture [38]. However, low forearm BMD is still clinically relevant because forearm BMD and consequent fracture also predict future risk of fracture [39]. Although BMD measurements at the hip and spine predict fractures better in the respective sites than forearm BMD [37], the latter measurement is easier to perform when technical or physical limitations are present (eg, prosthesis, arthritis, fractures, inability to lie still).

In addition to the main focus, the SNPs, a number of other potential modulating factors of BMD were included, and the expected associations were found. However, as this analysis was cross sectional, the causality of the associations must be carefully considered. For instance, the most probable explanation for the negative association between use of calcium and BMD is simply that patients with low BMD use calcium.

The present study has several limitations. First, our study may be biased due to population stratification as the study participants come from one geographical region. Though the SNPs of interest were chosen after associations were discovered in the other populations. Second, no information on some potential modulating factors of BMD, such as anorexia, recent hyperthyroidism, hypogonadism, oophorectomy, neurologic disease, recent immobilization, rickets, adrenal and renal bone disease or osteoporotic fractures, was available. Some data were based on self-administrated questionnaires with the inherent biases. Furthermore, BMD measurements of the forearm and hip were performed only once, and serum 25(OH)D and serum PTH measurements were from Tromsø 4 only.

The present study also has several strengths. The study was population-based. Due to previous genome-wide association studies (GWASs) and meta-analyses, we could focus on a limited number of SNPs. The serum 25(OH)D values were standardized and allowed comparison with other studies. Finally, the Mendelian randomization method, in which genetic variants are analyzed as proxies for lifelong differences in environmental exposure, is likely to minimize selection bias and to decrease the possibility of confounding [40,41].

In conclusion, we found an association between the serum 25(OH)D level-associated *CYP24A1* SNP rs6013897 and total hip BMD. However, the effects of this SNP on serum 25(OH)D levels and BMD were small and need confirmation in other studies, in particular in populations where vitamin D deficiency is prevalent. The *ESR1*-related SNP rs4870044 is associated with forearm and total hip BMD.

## Supporting information

S1 Table The supplemental baseline characteristics of the entire study population and subjects with valid BMD measurements of the forearm in Tromsø 4 and hip in Tromsø 5, and at least one successful SNP analysis*. The Tromsø Study.
(DOCX)

S2 Table Linear regression model for forearm BMD for women and men in Tromsø 4. (DOCX)

S3 Table Linear regression model for total hip BMD for women and men in Tromsø 5. (DOCX)

S4 Table Mean serum 25(OH)D values across the genotypes in Tromsø 4. (DOCX)
