# Supplementary material for: Bone mineral density is associated with vitamin D related rs6013897 and estrogen receptor polymorphism rs4870044: The Tromsø study
Source: PLoS One. 2017 Mar 2;12(3):e0173045. doi: 10.1371/journal.pone.0173045 (PMC5333870; doi:10.1371/journal.pone.0173045)
Supplement: S1 Table — The Tromsø Study. (DOCX) [file pone.0173045.s001.docx]

**S1 Table. The supplemental baseline characteristics of the entire study population and subjects with valid BMD measurements of the forearm in Tromsø 4 and hip in Tromsø 5, and at least one successful SNP analysis*. The Tromsø Study.**

|  | **Entire Tromsø 4 population**** | **Genotyped and BMD measured subjects in Tromsø 4** | **Entire Tromsø 5 population**** | **Genotyped and BMD measured subjects in Tromsø 5** |
| --- | --- | --- | --- | --- |
| N | 26,956 | 7,317 | 8,039 | 4,082 |
| Physical activity ≥1 h / week (% active) | 73.7 | 73.9 | 67.3 | 63.3 |
| Previous or current smoker (%)^a^ | 58.2 | 68.4 | 65.6 | 65.8 |
| Early menopause (%) | 30.2 | 28.2 | 28.3 | 27.7 |
| Self-reported cancer (%) | 3.5 | 6.0 | 7.5 | 8.5 |
| Self-reported diabetes (%) | 1.8 | 2.8 | 3.8 | 4.5 |
| Self-reported osteoarthritis (%) | 39.2 | 36.7 | 21.0 | 24.8 |
| Self-reported ulcer-related surgery (%) | 2.0 | 3.7 | 2.9 | 3.4 |
| Reported and registered current or previous use of drugs containing estrogen (%) | 8.4 | 13.3 | 17.9 | 21.8 |
| Registered use of systemic corticosteroids (%)^b^ | 0.3 | 1.0 | 0.4 | 2.0 |
| Registered use of drugs containing thiazide (%)^b^ | 1.3 | 1.3 | 2.2 | 2.6 |
| Reported and registered current or previous use of insulin (%) | 5.7 | 10.7 | 1.4 | 1.6 |
| Reported and registered current or previous use of other antidiabetic drugs than insulin (%) | 0.6 | 1.4 | 2.8 | 3.2 |
| Reported current use of vitamin D supplementation (cod liver oil or tablets) (%) | 13.9 | 35.5 | 43.1 | 57.6 |
| Reported current use of calcium supplementation (%) | 1.7 | 5.0 | 8.4 | 11.6 |
| Registered use of bisphosphonates (%)^b^ | 0.0 (N = 2) | 0.0 (N = 2) | 1.3 | 1.7 |

^a^In Tromsø 4, smoking status was defined by current or previous smoking status, while in Tromsø 5, smoking status was defined as current smoking daily or sometimes.

^b^In Tromsø 4, information attained only in those who attended the second visit in 1994–1995, N from 2,903 to 7,872.

* SNPs of interest include the SNPs presented in Table 2 (*FokI*, *Cdx2*, *BsmI*, rs2298850, rs10741657, rs3794060, rs6013897 and rs4870044).

**The number of subjects differs from the original number in description as the data in the table are presented based on the data available to the author.
