# Supplementary material for: Bone mineral density is associated with vitamin D related rs6013897 and estrogen receptor polymorphism rs4870044: The Tromsø study
Source: PLoS One. 2017 Mar 2;12(3):e0173045. doi: 10.1371/journal.pone.0173045 (PMC5333870; doi:10.1371/journal.pone.0173045)
Supplement: S2 Table — (DOCX) [file pone.0173045.s002.docx]

**S2 Table. Linear regression model for forearm BMD for women and men in Tromsø 4.**

|  | **Standardized beta coefficient** | ***P*–value** | **Standardized beta coefficient** | ***P*–value** |
| --- | --- | --- | --- | --- |
| **Covariates (all included in the regression model)** | **Women,**  **N = 1,674** |  | **Men,**  **N = 1,276** |  |
| Age (years)^a^ | –0.539** | <0.001 | –0.232** | <0.001 |
| BMI (kg/m^2^) | 0.232** | <0.001 | 0.253** | <0.001 |
| Height (cm) | 0.057** | 0.006 | 0.090** | 0.001 |
| Serum PTH (pmol/l)^b^ | –0.065** | 0.002 | –0.042 | 0.113 |
| Serum 25(OH)D (nmol/L)^b^ | 0.019 | 0.393 | –0.009 | 0.722 |
| Serum creatinine (μmol/L)^b^ | 0.090** | <0.001 | 0.047 | 0.071 |
| Serum calcium (mmol/L) | 0.053** | 0.008 | –0.013 | 0.607 |
| Physical activity ≥1 h/week (% active) | –0.006 | 0.765 | 0.018 | 0.483 |
| Previous or current smoker (%)^c^ | –0.041 | 0.051 | –0.069** | 0.008 |
| Early menopause (%) | –0.057** | 0.004 | NA | NA |
| Self-reported cancer (%) | –0.047* | 0.018 | –0.005 | 0.831 |
| Self-reported diabetes (%) | 0.016 | 0.532 | 0.005 | 0.897 |
| Self-reported osteoarthritis (%) | 0.017 | 0.459 | 0.020 | 0.464 |
| Self-reported ulcer-related surgery (%) | 0.001 | 0.946 | –0.116** | <0.001 |
| Reported and registered current or previous use of drugs containing estrogen (%) | 0.078** | <0.001 | NA | NA |
| Registered use of systemic corticosteroids (%)^b^ | –0.018 | 0.364 | –0.082** | 0.001 |
| Registered use of thiazide containing drugs (%)^b^ | –0.011 | 0.579 | 0.040 | 0.112 |
| Reported and registered current or previous use of insulin (%) | –0.040 | 0.099 | –0.078* | 0.010 |
| Reported and registered current or previous use of antidiabetic drugs other than insulin (%) | 0.018 | 0.465 | 0.012 | 0.753 |
| Reported current use of vitamin D supplementation (cod liver oil or tablets) (%) | 0.058** | 0.006 | -0.003 | 0.918 |
| Reported current use of calcium supplementation (%) | –0.071** | <0.001 | 0.022 | 0.387 |

^a^Age by the end of 1994 for Tromsø 4.

^b^In Tromsø 4, information attained only in those who attended the second visit in 1994–1995, N from 2,903 to 7,872.

^c^In Tromsø 4, the smoking status was defined by current or previous smoking status.

* *P* < 0.05 in the linear regression model. ** *P* < 0.01 in the linear regression model.

NA: not applicable. Adjusted R^2^ for the model 0.37 for women and 0.22 for men.
