# Supplementary material for: Bone mineral density is associated with vitamin D related rs6013897 and estrogen receptor polymorphism rs4870044: The Tromsø study
Source: PLoS One. 2017 Mar 2;12(3):e0173045. doi: 10.1371/journal.pone.0173045 (PMC5333870; doi:10.1371/journal.pone.0173045)
Supplement: S3 Table — (DOCX) [file pone.0173045.s003.docx]

**S3 Table. Linear regression model for total hip BMD for women and men in Tromsø 5.**

|  | **Standardized beta coefficient** | ***P*–value** | **Standardized beta coefficient** | ***P*–value** |
| --- | --- | --- | --- | --- |
| **Covariates (all included in the regression model)** | **Women,**  **N = 875** |  | **Men,**  **N = 626** |  |
| Age (years) | –0.352** | <0.001 | –0.102* | 0.012 |
| BMI (kg/m^2^) | 0.426** | <0.001 | 0.319** | <0.001 |
| Serum PTH (pmol/l)^a^ | –0.115** | <0.001 | 0.010 | 0.804 |
| Serum 25(OH)D (nmol/L)^a^ | 0.036 | 0.217 | 0.013 | 0.730 |
| Serum creatinine (μmol/L) | 0.020 | 0.495 | 0.004 | 0.912 |
| Serum calcium (mmol/L) | 0.003 | 0.915 | 0.014 | 0.707 |
| Physical activity ≥1 h/week (% active) | 0.083** | 0.007 | 0.084* | 0.027 |
| Previous or current smoker (%)^b^ | –0.073* | 0.010 | –0.112** | 0.003 |
| Early menopause (%) | –0.049 | 0.084 | NA | NA |
| Self-reported cancer (%) | –0.016 | 0.574 | –0.013 | 0.724 |
| Self-reported diabetes (%) | –0.024 | 0.522 | 0.021 | 0.748 |
| Self-reported osteoarthritis (%) | –0.024 | 0.417 | –0.010 | 0.792 |
| Self-reported ulcer-related surgery (%) | –0.038 | 0.176 | –0.111** | 0.003 |
| Reported and registered current or previous use of drugs containing estrogen (%) | –0.018 | 0.560 | NA | NA |
| Registered use of systemic corticosteroids (%) | 0.034 | 0.234 | 0.074 | 0.066 |
| Registered use of drugs containing thiazide (%) | 0.035 | 0.227 | 0.037 | 0.321 |
| Reported and registered current or previous use of insulin (%) | 0.051 | 0.123 | –0.094 | 0.052 |
| Reported and registered current or previous use of antidiabetic drugs other than insulin (%) | 0.032 | 0.388 | 0.131 | 0.084 |
| Reported current use of vitamin D supplementation (cod liver oil or tablets) (%) | 0.031 | 0.279 | 0.040 | 0.280 |
| Reported current use of calcium supplementation (%) | –0.024 | 0.412 | 0.013 | 0.718 |
| Registered use of bisphosphonates (%) | –0.076** | 0.009 | –0.078 | 0.057 |

^a^In Tromsø 4, information attained only for those who attended the second visit in 1994–1995, N from 2,903 to 7,872.

^b^In Tromsø 5, the smoking status was defined as current smoking daily or sometimes.

* *P* < 0.05 in the linear regression model. ** *P* < 0.01 in the linear regression model.

NA: not applicable. Adjusted R^2^ for the model 0.34 for women and 0.23 for men.
