# Supplementary material for: Bone mineral density is associated with vitamin D related rs6013897 and estrogen receptor polymorphism rs4870044: The Tromsø study
Source: PLoS One. 2017 Mar 2;12(3):e0173045. doi: 10.1371/journal.pone.0173045 (PMC5333870; doi:10.1371/journal.pone.0173045)
Supplement: S4 Table — (DOCX) [file pone.0173045.s004.docx]

**S4 Table. Mean serum 25(OH)D values across the genotypes in Tromsø 4.**

| **SNP** | **N** | **Major homozygote** | **Heterozygote** | **Minor homozygote** |
| --- | --- | --- | --- | --- |
| Rs2228570/rs10735810 (*FokI,* *VDR* SNP)** | 5723 | 53.5 ± 11..3 | 54.2 ± 11.8 | 54.7 ± 11.0 |
| Rs1544410 (*BsmI*, *VDR* SNP) | 5692 | 54.1 ± 11.3 | 53.9 ± 11.6 | 54.1 ± 11.6 |
| Rs731236 (*TaqI*, *VDR* SNP) | 5719 | 54.1 ± 11.3 | 53.9 ± 11.6 | 54.1 ± 11.7 |
| Rs7975232 (*ApaI*, *VDR* SNP) | 5708 | 53.9 ± 11.5 | 54.0 ± 11.6 | 54.1 ± 11.3 |
| Rs11568820 (*Cdx2*, *VDR* SNP) | 5710 | 54.0 ± 11.6 | 54.0 ± 11.3 | 53.9 ± 11.1 |
| Rs7968585 (*VDR* SNP) | 5694 | 54.1 ± 11.6 | 54.0 ± 11.5 | 54.0 ± 11.2 |
| Rs3782905 (*VDR* SNP) | 5691 | 54.1 ± 11.6 | 53.8 ± 11.4 | 54.5 ± 11.7 |
| Rs2239179 (*VDR* SNP) | 5715 | 54.1 ± 11.3 | 53.7 ± 11.6 | 54.3 ± 11.5 |
| Rs2298850 (*DBP* SNP)** | 5720 | 55.3 ± 11.8 | 52.4 ± 10.8 | 50.3 ± 10.4 |
| Rs10741657 (*CYP2R1* SNP)** | 5716 | 53.1 ± 11.1 | 54.5 ± 11.6 | 54.4 ± 11.8 |
| Rs3794060 (*NAD-SYN* SNP)** | 4116 | 54.5 ± 11.6 | 53.4 ± 11.3 | 53.2 ± 10.9 |
| Rs6013897 (*CYP24A1* SNP)** | 5698 | 54.4 ± 11.5 | 53.3 ± 11.4 | 53.4 ± 12.0 |

***** *P* < 0.05 in the linear regression model, adjusted for age, gender, and season (months, using dummy variables).

** *P* < 0.01 in the linear regression model, adjusted for age, gender, and season (months, using dummy variables).
